# Comparison of the survival outcomes of laparoscopic, abdominal and gasless laparoscopic radical hysterectomy for early-stage cervical cancer: trial protocol of a multicenter randomized controlled trial (LAGCC trial)

**DOI:** 10.3389/fonc.2023.1287697

**Published:** 2023-11-13

**Authors:** Xiaoyan Tang, Shan Zhou, Xuyin Zhang, Keqin Hua, Yuan He, Ping Wang, Yincheng Teng, Weiwei Feng

**Affiliations:** ^1^ Department of Gynecology, Obstetrics and Gynecology Hospital, Fudan University, Shanghai, China; ^2^ Office of Clinical Epidemiology, Obstetrics and Gynecology Hospital of Fudan University, Shanghai, China; ^3^ Department of Gynecology and Obstetrics, West China Second University Hospital, Sichuan University, Sichuan, China; ^4^ Department of Gynecology and Obstetrics, Shanghai Sixth People Hospital, Shanghai Jiaotong University, Shanghai, China; ^5^ Department of Gynecology and Obstetrics, Ruijin Hospital, School of Medicine, Shanghai Jiao Tong University, Shanghai, China

**Keywords:** cervical cancer, laparoscopic radical hysterectomy, gasless laparoscopic radical hysterectomy, abdominal radical hysterectomy, disease-free survival, overall survival, tumor-free principle

## Abstract

**Background:**

Radical hysterectomy (RH) is considered a cornerstone in the treatment of early-stage cervical cancer. However, the debate surrounding the optimal surgical approach, whether minimally invasive or open surgery, remains controversial. The objective of this trial is to evaluate the survival outcomes of cervical cancer patients who undergo different surgical approaches.

**Methods:**

This study is designed as a prospective, multicenter, open, parallel, and randomized controlled trial. A total of 500 patients diagnosed with stage IA1 with LVSI, IA2, IB1, or IB2 (2018 FIGO) will be recruited. Recruitment of participants started in November 2020. The participants will be randomly assigned to one of three groups: conventional laparoscopic RH, gasless laparoscopic RH, or abdominal RH. The primary endpoint of this trial is the 2-year disease-free survival (DFS) rate. The secondary endpoints will include the 2-year overall survival (OS) rate, 5-year DFS/OS, recurrence rates, operation time, intraoperative blood loss, surgery-related complications, and impact on quality of life (QoL).

**Discussion:**

We expect this trial to provide compelling and high-quality evidence to guide the selection of the most appropriate surgical approach for early-stage cervical cancer.

**Clinical trial registration:**

Chinese Clinical Trial Register, identifier ChiCTR2000035515.

## 1 Introduction

Cervical cancer ranks as the fourth most common cancer in women, with approximately 604,127 new cases in 2020 worldwide (1). Radical hysterectomy (RH) with pelvic lymphadenectomy is widely regarded as the standard treatment for early-stage cervical cancer. Since the introduction of laparoscopic radical hysterectomy (LRH) in the 1990s (2), minimally invasive surgery (MIS), whether performed via laparoscopy or robotic surgery, has gained widespread acceptance as an alternative to abdominal radical hysterectomy (ARH) due to its various benefits, including reduced morbidity, shorter hospital stays, and quicker recovery times (3–6). A number of retrospective studies have reported the feasibility, advantages, and oncologic safety of the MIS approach (7–9). Moreover, the application of ultra-minimally invasive surgery for gynecological procedures was further explored, and showed good feasibility and safety (10). However, the famous Laparoscopic Approach to Carcinoma of the Cervix (LACC) trial in 2018, a multicenter, randomized, controlled trial (RCT), demonstrated that MIS was associated with lower rates of disease-free survival and overall survival compared to open surgery in women with early-stage cervical cancer (11). A similar finding was obtained in a retrospective analysis using the National Cancer and Surveillance, Epidemiology, and End Results (SEER) data (12). Consequently, guidelines such as those provided by the National Comprehensive Cancer Network (NCCN) and the European Society of Gynecological Oncology (ESGO) have recommended the open abdominal approach as the “standard and recommended approach to radical hysterectomy” (13, 14).

However, it should be acknowledged that the LACC trial also has some weaknesses (15). For instance, the survival rate of the open surgery group in the LACC trial was too high, much higher than that reported previously. Patients with recurrence were concentrated in 14 out of 33 centers, implying the presence of quality deviations among different centers in the trial. In contrast, reports from some recently published retrospective studies and meta-analyses have suggested that MIS is not inferior to open surgery for treating early-stage cervical cancer (16–19). Notably, in the subgroup analysis of the LACC trial, no survival difference was found between MIS and open surgery for patients with stage IB1 (<2 cm) (20). This finding has been corroborated by several other retrospective studies (21). Consequently, the need for additional high-quality RCTs is evident.

Certain hypotheses have been proposed to explain the poorer prognosis associated with MIS, including the use of a uterine manipulator, intracorporeal colpotomy, and the potential spillage of cells during pelvic lymphadenectomy (15). Notably, an observational study of 693 patients found that MIS with protective vaginal closure resulted in oncologic outcomes similar to those of open surgery (hazard ratio (HR): 0.63, 95% confidence interval (CI): 0.15–2.59) (21). In the Atsushi et al. study, they observed that the 3-year DFS rates (NLNT 92.4%; ARH 94.0%) and overall survival rates did not differ significantly between the MIS and open surgery groups by using the no-look no-touch (NLNT) technique, which included creation of the vaginal cuff, bagging the specimen and proceeding without an intra-uterine manipulator (22). Nevertheless, the debate regarding whether oncologic outcomes of MIS, with modifications to prevent tumor spillage, are truly equivalent to those of open surgery remains ongoing.

Additionally, it has been suggested that carbon dioxide (CO_2_) pneumoperitoneum during MIS can stimulate and proliferate tumor cells that spill into the peritoneal cavity. A retrospective analysis demonstrated that total laparoscopic/robotic intracorporeal colpotomy under CO_2_ pneumoperitoneum may carry a risk of a positive vaginal cuff margin, as well as intraperitoneal tumor spreads during MIS (23). Gasless laparoscopy, which was initially developed to overcome clinical and financial challenges associated with pneumoperitoneum and general anesthesia, has been successfully applied in various gynecological and gastrointestinal surgeries (24–26). Many trials have demonstrated the safety and efficiency of gasless laparoscopy (27, 28). However, its potential application in the treatment of cervical cancer and its effect on prognosis warrant further investigation.

In the current study, we aim to conduct a multicenter RCT to compare the oncologic outcomes of early-stage cervical cancer patients (stage IA1 with LVSI, IA2, IB1, IB2) who undergo conventional laparoscopic, gasless laparoscopic or abdominal radical hysterectomy with modifications of technique such as a protective colpotomy and elimination of uterine manipulator usage. Additionally, we will compare the quality of life (QoL), adverse events and surgery-related complications among the different surgical approaches. Furthermore, we will investigate the risk factors affecting the prognosis of cervical cancer in this study. We hope to provide valuable insights into the most effective and safe surgical approach for early-stage cervical cancer and contribute to ongoing efforts to improve patient outcomes and enhance the overall management of the disease.

## 2 Methods

### 2.1 Study design

**Figure 1:**
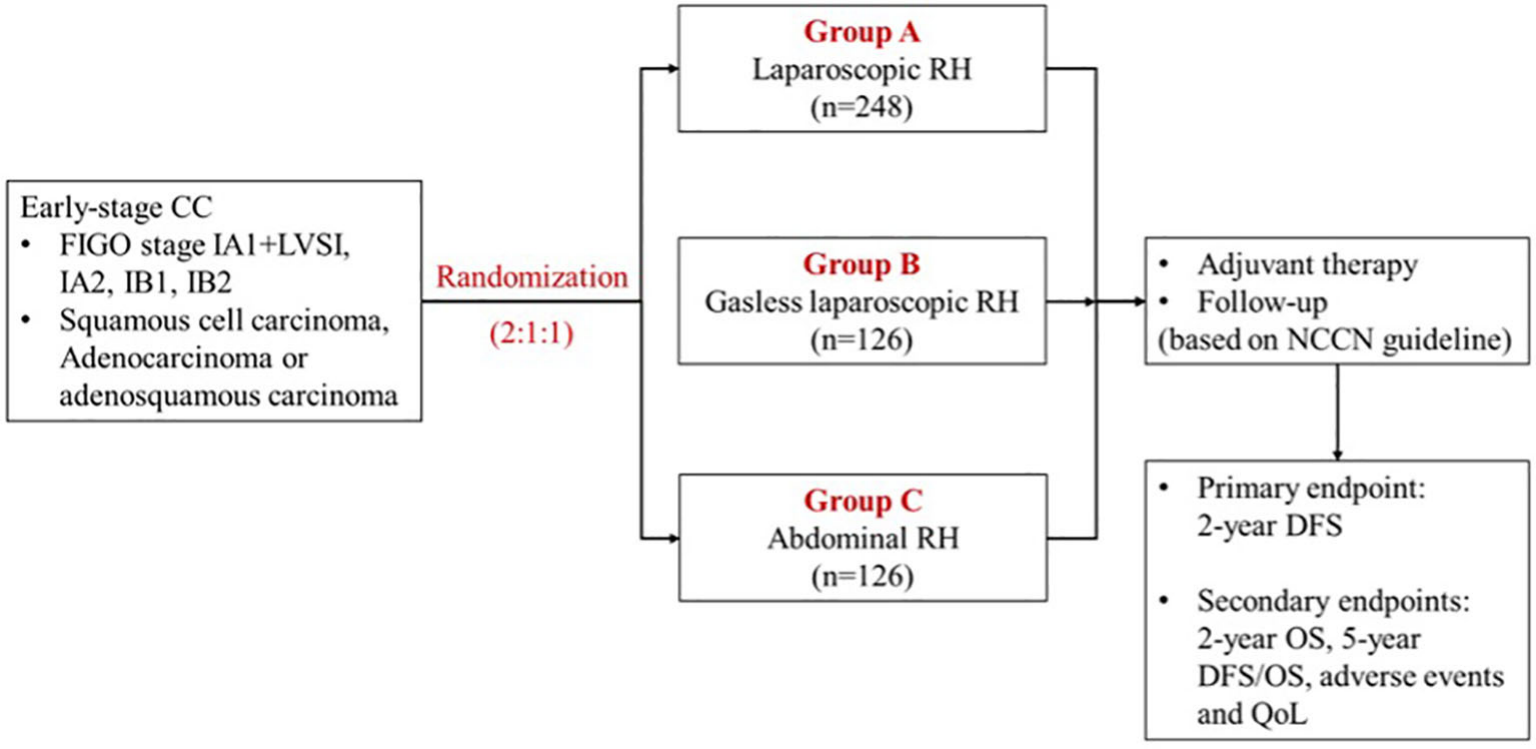
Trial scheme. CC, cervical cancer; FIGO, International Federation of Gynecology and Obstetrics; RH, radical hysterectomy; NCCN, National Comprehensive Cancer Network; DFS, disease-free survival; OS, overall survival; QoL, quality of life.

This prospective, multicenter, open, parallel, randomized controlled study was mainly focused on investigating the prognosis of early-stage cervical cancer patients who underwent surgical treatments with different approaches. Participants are randomly assigned to receive conventional LRH, gasless LRH or ARH (**Figure 1**). The trial will be conducted in the leading center, Obstetrics and Gynecology Hospital of Fudan University, and the other 3 participating centers in China: Shanghai Sixth People’s Hospital, West China Second University Hospital of Sichuan University and Ruijin Hospital Affiliated to Shanghai Jiaotong University School of Medicine. All four hospitals are Class A tertiary hospitals in China equipped with advanced medical technologies, multidisciplinary health care teams, and robust research capabilities, enabling them to offer comprehensive services related to cervical cancer. The study is registered at http://www.chictr.org.cn/(ChiCTR2000035515, Registered 13 August 2020). Recruitment of participants started in November 2020 and the last participant is expected to reach the primary endpoint (2-year follow-up) in November 2025.

### 2.2 Participants

#### 2.2.1 Inclusion criteria

Participants meeting all of the following criteria will be considered for enrollment: (1) clinical diagnosis of early stage of cervical cancer (stage IA1 with LVSI, IA2, IB1, IB2, IIA1, 2018 FIGO); (2) histologically confirmed squamous carcinoma, adenocarcinoma or squamous adenocarcinoma of the cervix; (3) age ≥18 years and ≤ 70 years; (4) no history of other malignancies; (5) not pregnant; (6) physical strength classification-Karnofsky score ≥ 60; (7) voluntary agreement to participate in the study, sign the informed consent form, and demonstrate good compliance with follow-up. The tumor stage will be confirmed after independent examination by two senior gynecologic oncologists based on the pathology report and pelvic MRI/CT results. The diagnosis of IA1 with LVSI and IA2 will require the pathology report from the loop electrosurgical excision procedure (LEEP) or conization.

#### 2.2.2 Exclusion criteria

Participants meeting any of the following criteria will be excluded (1): contraindicated for various surgical procedures (2); have received pelvic/abdominal radiotherapy irradiation or neoadjuvant chemotherapy for cervical cancer (3); recurrent cervical cancer (4); CT, MRI or PET-CT suggesting suspicious metastasis of lymph nodes or distant metastasis.

### 2.3 Endpoints

The primary endpoint is 2-year disease-free survival (DFS). The secondary endpoints include the 2-year overall survival (OS), 5-year DFS/OS, operation time, intraoperative blood loss, surgery-related complications, and quality of life (QoL). DFS and OS will be defined as the interval from the time of surgery to the time of recurrence or death for any reason, respectively. Recurrence will be diagnosed based on radiographic evidence using the Response Evaluation Criteria in Solid Tumors (RECIST) criteria. The EORTC-QLQ-C30 V3.0 questionnaire will be applied to assess the quality of life (QoL).

### 2.4 Randomization

Randomization will be conducted using an online computer algorithm to allocate the radical hysterectomy (RH) approach at a ratio of 2:1:1 (laparoscopy vs. laparotomy vs. gasless laparoscopy group). The computer algorithm will generate a unique code for each participant and collect basic information in addition to group assignment. Patients and study surgeons will be aware of the treatment assignment.

### 2.5 Qualification requirements for the chief surgeon

The chief surgeons are carefully selected from each participating hospital and had expertise in cervical cancer surgeries. They must have performed a minimum of 50 surgeries for both LRH and ARH, with documented case histories. Each surgeon is required to submit unedited surgical videos (1 ARH and 1 LRH) for the principal investigator and the surgical quality control team to review to ensure precise surgical resection and tumor-free management.

### 2.6 Treatments

**Figure 2:**
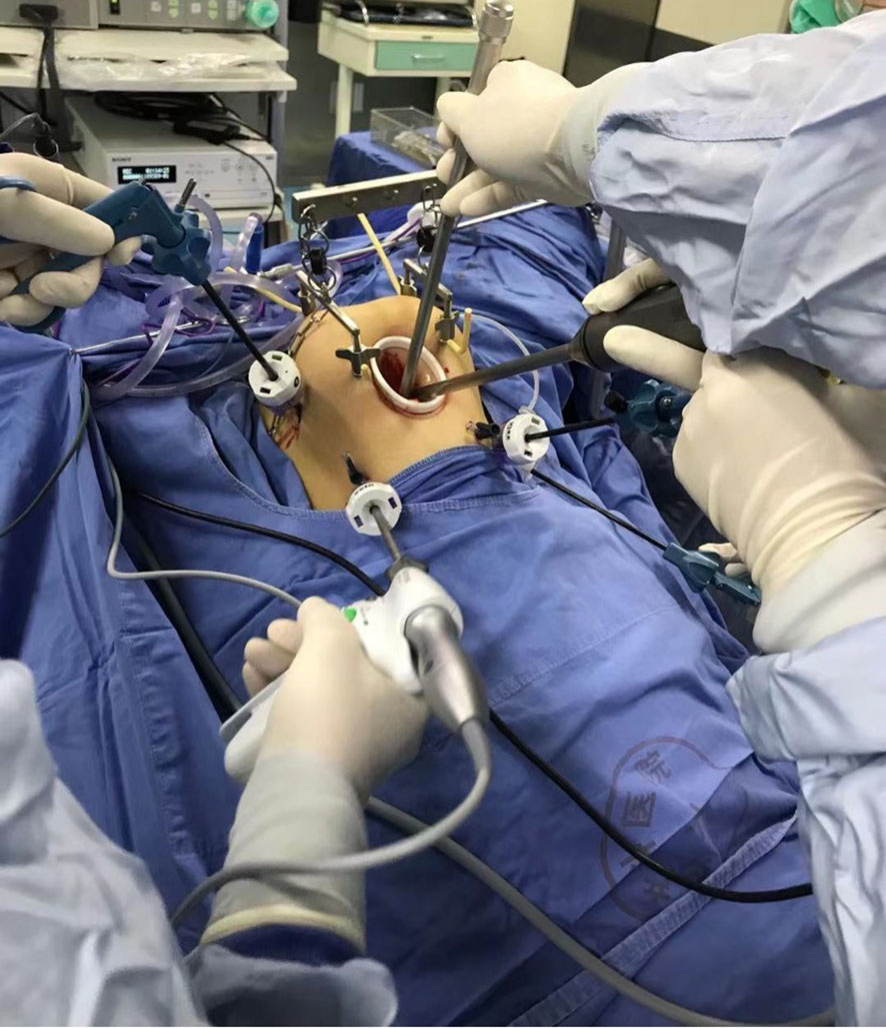
Gasless laparoscopy was performed with abdominal wall suspension.

For the gasless LRH procedure, a combination of special surgical instruments (**Figure 2**) and conventional laparoscopic instruments was utilized. The process involved the following steps: After inducing anesthesia, the patient was positioned in a lithotomy position, and a sterilized stainless-steel scaffold with a lifting arm was attached to the operating table. Two sterilized needles were inserted through the subcutaneous tissue of the left and right abdomen to lift the abdominal wall and provide pelvic exposure. A 2 cm mini-laparotomy incision was made, and a small Alexis wound protector was used to facilitate smooth entry of the 10 mm laparoscope. The patient was adjusted to approximately 30° in the Trendelenburg position. Four 5 mm conventional laparoscopic access ports were inserted: one at McBurney’s point, one at the anti-McBurney point, and two fingers (3–4 cm) above the umbilicus in the left and right medio clavicular lines.

Patients underwent different surgical approaches according to group while maintaining the same surgical scope based on tumor stage as follows:

1) Patients with IA1 (LVSI+) and stage IA2 underwent modified radical hysterectomy (type B), while IB1, IB2, and IIA1 patients underwent radical hysterectomy (type C) according to the new classification of RH proposed by Querleu and Morrow (29).
2) Systematic pelvic lymphadenectomy was performed, including the removal of lymph nodes adjacent to the common iliac artery, external iliac artery, internal iliac artery, and obturator fossa. Para-aortic lymph node dissection was performed where necessary, and after dissection on one side, the lymph nodes were bagged and sealed separately.
3) A strict adherence to the tumor-free principle was followed, where the vagina was closed before severing it. This could be accomplished through the closure of the vagina with an obturator, forceps, a ligature ring, and transvaginal sutures during laparoscopic surgery or using an open surgical kidney pedicle during open surgery. The use of any type of uterine manipulator was strictly prohibited during the surgery.
4) The ovaries may be preserved or removed depending on the patient’s condition. For patients who are younger than 45 years old, without signs of ovarian metastasis from both preoperative imaging examination and intraoperative findings, ovarian preservation will be considered. If the ovaries are preserved, ovarian transposition is recommended to avoid potential damage from subsequent radiotherapy.

Patients with high-risk factors, such as positive resection margins, lymph node metastasis, and parametrial involvement, are expected to receive adjuvant therapy. Additionally, for patients with cervical squamous cell carcinoma who meet the Sedlis criteria, adjuvant therapy is also encouraged. Postoperative adjuvant treatment will be administered in accordance with the latest NCCN guidelines.

Standardized postoperative follow-up will be strictly conducted based on the NCCN guideline, with visits scheduled every 3 months for 2 years, every 6 months for the following 3 years, and annually thereafter (**Table S1**). Routine follow-up items will include physical examinations, HPV tests, LCT, ultrasound, enhanced pelvic MRI, PET-CT, and colposcopy when deemed necessary. Moreover, detailed information on complications arising from both surgeries and adjuvant therapy will also be collected during the follow-up period.

## 3 Statistical analysis

### 3.1 Sample size

The primary objective of this study was to compare oncologic outcomes among patients undergoing gasless LRH, ARH, and conventional LRH, focusing on 2-year disease-free survival (DFS) as the primary endpoint. Based on previous research results, the estimated 2-year DFS rates for patients undergoing gasless LRH, ARH, and LRH were 97%, 98%, and 96%, respectively. For the study, the noninferiority margin was set at 9%, and there will be a follow-up period of 2 years.

Considering the relative complexity of performing gasless LRH in participant centers compared to leading centers, the sample ratio was set at 2:1:1 (laparoscopy vs. laparotomy vs. gasless laparoscopy group) for statistical analysis, using a one-sided test with a significance level (α) of 0.025. Additionally, a 10% dropout rate, including those lost to follow-up, was assumed for the study. Based on these considerations, the study is estimated to have a statistical power of 97% to detect any survival differences (one-sided test, α=0.025).

In summary, considering the maximum sample size, both the laparotomy group and the gasless laparoscopy group will each require 126 patients, while the laparoscopy group will require 248 patients, resulting in a total of 500 patients needed for the study.

### 3.2 Data management

For each admitted case, a clinical trial electronic case report form (eCRF) completed by the investigators will be used to record and deposit patient data. The collected data will be reviewed by the investigators and handed over to the data administrators for entry and management. To ensure data accuracy, two data administrators will independently perform double entry, followed by computer verification and manual cross-checking. The data will then be handed over to statisticians for blind verification and subsequent statistical analysis. This comprehensive data management process will ensure the integrity and reliability of the data used in the study.

### 3.3 Data analysis

#### 3.3.1 Analysis of primary endpoints

Primary endpoints will be analyzed according to the intention-to-treat principle. Missing data will be censored at the last known date of patient survival. Sensitivity analysis will be performed based on the per-protocol (PP) principle. The study is intended to assess the noninferiority of the 2-year disease-free survival (DFS) rate in the LRH group compared to the ARH and gasless LRH groups, with a noninferiority limit set at 9%.

The DFS rate will be estimated using Kaplan-Meier curves, while the hazard ratio (HR) and one-sided 97.5% confidence interval (CI) will be calculated using a Cox proportional hazards regression model, confirming the proportional hazard assumption. One-sided 97.5% CIs will be reported for DFS differences and HRs, whereas other CIs will be two-sided 95% CIs unless otherwise specified.

#### 3.3.2 Analysis of secondary endpoints

(1) The 2-year OS, 5-year DFS/OS will be estimated using Kaplan-Meier curves and compared between groups by log-rank tests or the Cox proportional hazards regression model.
(2) The frequency of surgery-related complications will be analyzed using the χ2 test.
(3) To compare discrepancies in QoL scores and other continuous variables, including biochemical indicators, a T test or Mann-Whitney test will be employed. Categorical variables, such as imaging markers, were analyzed using the χ2 test or logistic regression.
(4) The rates of adverse events will be compared using the χ2 test or Fisher’s exact test.
(5) Univariate and multivariate Cox regression will be used to explore risk factors for OS and DFS rates in patients with different surgical approaches. Variables of interest will include tumor size, FIGO stage, and pathological characteristics (e.g., stromal invasion depth, lymph-vascular space invasion (LVSI), and lymph node metastasis).

## 4 Ethics and dissemination

The protocol was approved by the Ethics Committees of Obstetrics and Gynecology Hospital of Fudan University (2020–159) and the other 3 participating centers. The protocol version number and date were 1.0 and 25 October 2020. The trial will be conducted in accordance with the principles of the World Medical Association’s Declaration of Helsinki and adhere to Good Clinical Practice (GCP) standards.

## 5 Discussion

Since the publication of the LACC trial and retrospective analysis based on SEER data, the NCCN guideline (version 3.2019) have recommended the open abdominal approach as the standard approach for radical hysterectomy (13). However, despite the RCT design of the LACC trial, there are some limitations in its methodology. Recent studies have produced contradictory findings, supporting the noninferiority of LRH compared to ARH. However, most of these studies were retrospective or meta-analyses, and high-quality RCTs are still lacking. Therefore, the debate on LRH versus ARH continues, and there is a need for high-quality evidence to guide clinical practice.

In light of the inconsistencies in various studies, it is crucial to identify the underlying causes contributing to the differences in disease recurrence. One potential factor is tumor spillage caused by intracorporeal colpotomy and the use of a uterine manipulator during laparoscopy, which has been implicated in disease recurrence and survival outcomes in MIS for cervical cancer. This factor was not considered in the LACC trial. Moreover, the proficiency of surgeons has also been shown to influence the survival outcomes of MIS (30). The surgeon criterion for LRH in the LACC trial was only 10 cases, which is definitely insufficient.

Additionally, the high recurrence rate of MIS may be related to the use of CO_2_ (31). Studies have confirmed that CO_2_ can promote tumor growth in nude mice and increase the risks of metastases in the abdominal wall incision, peritoneal implantation, and dissemination (32, 33). To address this concern, we developed a gasless laparoscopy approach for performing radical hysterectomy for early cervical cancer with abdominal wall suspension. This technique eliminates the effect of CO_2_ pneumoperitoneum, harnesses the advantages of MIS, and aims to achieve outcomes comparable to those of laparotomy.

In this trial, we enrolled patients with stages IA1 with LVSI, IA2, IB1, IB2, and IIA1, as surgery is the preferred treatment for these patients. The patients will be randomly assigned to undergo LRH, gasless LRH, or ARH in a 2:1:1 ratio. To ensure surgery quality, we have established strict criteria. Participating surgeons are required to have experience in at least 50 operations of LRH and ARH each. The above-mentioned tumor-free principle, as described in the Methods section, must be strictly adhered to during LRH, gasless LRH, and ARH procedures. We aimed to evaluate the oncologic outcomes of patients with early-stage cervical cancer undergoing different surgical approaches with detailed technical improvements. If LRH is indeed associated with a poorer prognosis than ARH, we will assess whether gasless laparoscopy, a surgical procedure that theoretically simulates the environment of open surgery while combining the advantages of laparoscopy and laparotomy, can be noninferior to open surgery.

In addition, there is an increasing focus on the safety of minimally invasive approaches in “low-risk” early-stage cervical cancer. The results of the LACC trial were not powered to evaluate the difference in oncologic outcomes of LRH and ARH for patients with “low-risk” cervical cancer. He et al. reported that 5-year OS (96.9% vs. 97.3%, p=0.44) and 5-year DFS (94.5% vs. 95.0%, p=0.22) were similar between LRH and ARH for patients with tumors <2 cm in a multicenter retrospective cohort study in 37 hospitals in China (34). Similar findings were obtained in Chen et al.’s study, which focused on “low-risk” cervical cancer patients with a tumor size<2 cm, no LVSI, superficial stromal invasion, and no lymph node involvement on imaging (35). However, a multi-institutional retrospective review revealed that MIS was associated with higher recurrence, even in patients with a tumor size ≤2 cm (36). Considering that the relatively short-term follow-up might influence the interpretation of the results in this “low-risk” group, a recently published propensity-score based analysis revealed that for low-risk patients, LRH does not result in worse 10-year outcomes than the open approach (37). In this study, we will also try to explore the appropriate approach for “low-risk” early-stage cervical cancer in the subgroup analysis. Considering the distinct biological behavior and prognosis between cervical adenocarcinoma and squamous cell carcinoma (38), we also investigated risk factors including histology, tumor size, FIGO stage, and other pathological characteristics, for the decrease in OS and PFS rate of patients with different surgical approaches. Furthermore, it has been reported that the recurrence pattern differs among cervical cancer patients undergoing LRH or ARH, and the risk factors for recurrence varies in different studies (15, 39–41). In this study, we will also investigate the recurrence risk factors and patterns associated with different surgical approaches.

The limitations of this study are as follows: first, the relatively innovative surgical method of gasless laparoscopy may make surgery more difficult. However, all the participating chief surgeons have expertise in radical surgeries for cervical cancer. The relevant indexes of gasless LRH, including perioperative bleeding, operation time, hospital stay, and complications during and after surgery were comparable to those of conventional LRH in our previous small-size study. Second, 2-year DFS instead of 5-year DFS was used for the primary endpoint, although it is not conventionally used as a trial endpoint. However, using a 2-year DFS, we can attain a primary endpoint with minimal follow-up period, and 5-year DFS/OS will also be analyzed as secondary endpoints.

In conclusion, we hope that our RCT will provide more precise and convincing evidence to assist gynecologic oncologists and patients in selecting the appropriate surgical approach. Moreover, the trial’s evaluation of secondary endpoints, such as recurrence rates, operation time, intraoperative blood loss, surgery-related complications, and QoL, will enhance our understanding of the impact of different surgical approaches. The comprehensive findings will inform medical practitioners regarding the provision of treatments that optimize survival and postsurgical quality of life for early-stage cervical cancer patients. Ultimately, this research is expected to shape clinical practices, improve patient outcomes, and advance cervical cancer treatment standards.

## Data availability statement

The original contributions presented in the study are included in the article/**Supplementary Material**. Further inquiries can be directed to the corresponding authors.

## Ethics statement

The studies involving humans were approved by Ethics Committees of Obstetrics and Gynecology Hospital of Fudan University (2020–159). The studies were conducted in accordance with the local legislation and institutional requirements. Written informed consent for participation in this study was provided by the participants’ legal guardians/next of kin.

## Author contributions

XT: Methodology, Visualization, Writing – original draft, Writing – review & editing. SZ: Writing – original draft, Writing – review & editing, Investigation. KH: Conceptualization, Investigation, Methodology, Project administration, Supervision, Writing – review & editing. XZ: Conceptualization, Investigation, Methodology, Project administration, Supervision, Writing – review & editing. YH: Methodology, Writing – review & editing. PW: Investigation, Writing – review & editing. YT: Investigation, Writing – review & editing. WF: Investigation, Writing – review & editing.
