# Supplementary material for: Comparison of the survival outcomes of laparoscopic, abdominal and gasless laparoscopic radical hysterectomy for early-stage cervical cancer: trial protocol of a multicenter randomized controlled trial (LAGCC trial)
Source: Front Oncol. 2023 Nov 13;13:1287697. doi: 10.3389/fonc.2023.1287697 (PMC10679326; doi:10.3389/fonc.2023.1287697)
Supplement: Supplementary file 1 [file Table_1.docx]

| Postoperative time point (month) | Physical examination | LCT | HPV | Biomarkers  (SCCA, CA125, CA199, HE4, CEA) | Imaging | | Oncologic outcomes  (Recurrence and survival) | QoL | Adverse events |
| --- | --- | --- | --- | --- | --- | --- | --- | --- | --- |
|  |  |  |  |  | **Ultrasound** | **CT, enhanced pelvic MRI**  **(PET-CT when necessary)** |  |  |  |
| 1 | √ |  |  | √ | √ |  |  | √ | √ |
| 3 | √ | √ | √ | √ | √ |  | √ |  | √ |
| 6 | √ | √ | √ | √ | √ |  | √ | √ | √ |
| 9 | √ |  |  | √ | √ |  | √ |  | √ |
| 12 | √ | √ | √ | √ | √ | √ | √ | √ | √ |
| 15 | √ |  |  | √ | √ |  | √ |  | √ |
| 18 | √ | √ | √ | √ | √ |  | √ |  | √ |
| 21 | √ |  |  | √ | √ |  | √ |  | √ |
| 24 | √ | √ | √ | √ | √ | √ | √ | √ | √ |
| 30 | √ |  |  | √ | √ |  | √ |  | √ |
| 36 | √ | √ | √ | √ | √ | √ | √ | √ | √ |
| 42 | √ |  |  | √ | √ |  | √ |  | √ |
| 48 | √ | √ | √ | √ | √ | √ | √ | √ | √ |
| 54 | √ |  |  | √ | √ |  | √ |  | √ |
| 60 | √ | √ | √ | √ | √ | √ | √ | √ | √ |

Table S1. Postoperative follow-up schedule

LCT, liquid-based cytology test; HPV, human papillomavirus; SCCA, squamous cell carcinoma antigen; CA, cancer antigen; HE4, human epididymis protein 4; CEA, carcinoembryonic antigen; CT, computed tomography; MRI, magnetic resonance imaging; PET-CT, positron emission tomography-computed tomography; quality of life.
